# Proteomic analysis of the anti-inflammatory action of minocycline

**DOI:** 10.1002/pmic.201000273

**Published:** 2010-12-06

**Authors:** Christopher R Dunston, Helen R Griffiths, Peter A Lambert, Susan Staddon, Ann B Vernallis

**Affiliations:** 1 Life and Health Sciences, Aston UniversityAston Triangle, Birmingham, UK; 2 Oxford Radcliffe Hospitals NHS trustOxford, UK

**Keywords:** 2-DE, Cell biology, Lipopolysaccharide, Macrophage, Minocycline, Tigecycline

## Abstract

Minocycline possesses anti-inflammatory properties independently of its antibiotic activity although the underlying molecular mechanisms are unclear. Lipopolysaccharide (LPS)-induced cytokines and pro-inflammatory protein expression are reduced by minocycline in cultured macrophages. Here, we tested a range of clinically important tetracycline compounds (oxytetracycline, doxycycline, minocycline and tigecycline) and showed that they all inhibited LPS-induced nitric oxide production. We made the novel finding that tigecycline inhibited LPS-induced nitric oxide production to a greater extent than the other tetracycline compounds tested. To identify potential targets for minocycline, we assessed alterations in the macrophage proteome induced by LPS in the presence or absence of a minocycline pre-treatment using 2-DE and nanoLC-MS. We found a number of proteins, mainly involved in cellular metabolism (ATP synthase β-subunit and aldose reductase) or stress response (heat shock proteins), which were altered in expression in response to LPS, some of which were restored, at least in part, by minocycline. This is the first study to document proteomic changes induced by minocycline. The observation that minocycline inhibits some, but not all, of the LPS-induced proteomic changes shows that minocycline specifically affects some signalling pathways and does not completely inhibit macrophage activation.

## 1 Introduction

The tetracycline family of compounds are widely used as broad-spectrum antibiotics. The recent studies have identified a number of non-antibiotic effects that are shared by most tetracycline compounds. These include anti-oxidant 1, anti-apoptotic 2, anti-metastatic 3 and most notably anti-inflammatory effects 2, 4–6. Tetracyclines are inexpensive, have a very good safety profile and have been investigated in the treatment of a number of inflammatory diseases including rheumatoid arthritis 6, 7, periodontitis 8, cardiac ischaemia–reperfusion injury 9 and a wide range of central nervous system pathologies including Parkinson's disease 10, multiple sclerosis 11, amylotrophic sclerosis 12, 13, and both global and focal cerebral ischaemia 4, 5.

The molecular mechanism(s) underpinning the anti-inflammatory action of tetracyclines is currently unclear but is at least in part due to the inhibition of activation of macrophages 6, 14 and macrophage-like cells such as microglia in the CNS 15. Tetracyclines are able to inhibit lipopolysaccharide (LPS)-induced activation of macrophages and microglia both in vitro and ex vivo. This is characterised by the inhibition of the production and release of pro-inflammatory cytokines such as tumour necrosis factor α, interleukin 1β and interleukin 6 15 and pro-inflammatory proteins such as inducible nitric oxide synthase (iNOS) 6, 14, cyclo-oxygenase 2 16 and matrix metalloproteinases 17. More recently, minocycline has been shown to be a potent inhibitor of poly-(ADP)ribose polymerase by acting as a mimetic of the essential co-factor, NADH 18, and secretory phospholipase A2 19.

LPS is a component of the cell wall of Gram-negative bacteria and is the dominant microbial component that is recognized by the mammalian innate immune system to initiate an immune response. The use of LPS in experimentation is widespread and is typically used as a model of inflammation. LPS is either spontaneously shed from the bacterium whereby it is sequestered by the mammalian serum protein, LPS-binding protein (LBP), alternatively LBP can directly extract LPS from micelles or directly from the bacterium itself 20. LBP then presents the monomeric LPS to CD14 that is expressed on the cell surface of many cells of the innate immune system. CD14 in turn delivers the LPS molecule to the toll-like receptor (TLR4) receptor via the cell membrane-bound adaptor molecule MD-2. TLR4 homodimerisation follows LPS ligation initiating the intracellular recruitment of a number of TLR4 adaptors including Mal and MyD88 21. MyD88-dependent signalling is transduced via the activation of a number of signalling proteins including TRAF6, IRAK and TAK1. MyD88-dependent LPS signalling culminates in the activation of mitogen-activated protein kinase pathways and activation nuclear factor (NF)-κB 22. Nitric oxide is produced from the conversion of arginine to nitric oxide (NO^•^) and citrulline, a reaction catalysed by the enzyme iNOS. iNOS is not expressed in the resting macrophage and is induced by LPS. Transcription of iNOS is regulated by NF-κB and AP-1 transcription factors 23, both of which are required for full and complete transcriptional activation of the iNOS gene. Nitric oxide has been implicated in the pathogenesis of many inflammatory diseases including rheumatoid arthritis 24 and sepsis 25.

In this study, we have used 2-DE and nano-LC-MS to identify for the first time alterations in the macrophage proteome in response to LPS with or without the addition of minocycline to investigate the mechanisms of its anti-inflammatory activity.

## 2 Materials and methods

### 2.1 Chemicals and reagents

All chemicals were purchased from Sigma-Aldrich (Poole, UK) unless otherwise stated. Oxytetracycline hydrochloride, doxycycline hyclate and minocycline hydrochloride were purchased from Sigma-Aldrich, tigecycline was purchased from AAH pharmaceuticals (Coventry, UK). Anti-iNOS antibody (sc-650) was purchased from Santa-Cruz Biotechnology (Santa-Cruz, USA). Anti-β-actin antibody (ab-6276) was purchased from AbCam (Cambridge, UK).

### 2.2 Cell culture

The J774.2 murine macrophage cell line was cultured in DMEM supplemented with 2 mM glutamine (Invitrogen; Paisley, UK), streptomycin 100 μg/mL, penicillin 100 U/mL (Invitrogen) and 10% heat-inactivated FBS (Lonza) and maintained at 37°C in an atmosphere of 5% CO_2_ and 95% air.

### 2.3 Cytotoxicity assay

Cytotoxicity was assessed by the cells' ability to reduce 3-(4,5-dimethylthiazol-2-yl)-2,5-diphenyltetrazolium (MTT) 26. Cells were cultured in 100 μL DMEM with 10% FBS and stimulated with either oxytetracycline, doxycycline, minocycline or tigecycline, after 1 h LPS (1 μg/mL) was added for 24 h. After 24 h 25 μL of MTT solution (5 mg/mL in PBS) was added and the cells were incubated at 37°C for 4 h, cells were then lysed with lysis buffer (20% w/v SDS in 50% DMF and 2.5% acetic acid) and incubated at 37°C for 16 h. After the incubation with lysis buffer, the absorbance of the wells was measured at 570 nm.

### 2.4 Nitrite determination

Cells were seeded in 6-well plates overnight, cells were then dosed with either oxytetracycline, doxycycline, minocycline or tigecycline, LPS was then added, after 24 h cell culture supernatant was removed, centrifuged at 12 000 rcf for 5 min to remove cell debris and cell culture supernatants were stored at −80°C until analysd. Sulphanilamide (50 μL; 1% w/v sulphanilamide in 5% v/v phosphoric acid) was added to 50 μL cell culture media and incubated at room temperature in the absence of light for 10 min, 50 μL of 0.1% w/v *N*-1-naphthylethylenediamine dihydrochloride was added to each of the samples and standards and the plate was then incubated for a further 10 min in the absence of light. Absorbance was then measured at 550 nm with an Anthos 2001 plate reader. Unknown concentrations of nitrite were calculated by constructing a standard curve using known concentrations of sodium nitrite.

### 2.5 Immunoblotting

Cells were suspended in denaturing buffer (10% v/v glycerol, 2% w/v SDS, 0.007 w/v bromophenol blue, 63 mM Tris–HCl, 2 mM sodium pyrophosphate, 5 mM EDTA, 50 mM DTT). The suspensions were drawn up and down a 21G needle to sheer genomic DNA and then heated to 95°C for 5 min. Protein content was determined using the RC DC protein assay (BioRad) according to the manufacturer's instructions. Equal amounts of protein (20 μg) were separated by SDS-PAGE on 7.5% w/v acrylamide gels, electro-transferred to Hybond nitrocellulose membrane (GE Healthcare, Amersham, UK) and blocked in 3% BSA in Tris-buffered saline with 0.1% Tween20 (TTBS) for 2 h. Following blocking the nitrocellulose was incubated with the primary antibody (anti-iNOS (1:1000) or anti-actin (1:10 000) in TTBS with 0.2% BSA overnight at room temperature. Following the primary antibody incubation, the nitrocellulose membrane was washed and the respective secondary antibody (1:10 000) was suspended in TTBS with 0.2% BSA and placed onto the membrane for 90 min. After washing the nitrocellulose the protein on the membrane was detected with ECL reagent (GE Healthcare) and Hyperfilm (GE Healthcare).

### 2.6 2-DE

Cells were suspended in rehydration buffer (8 M urea, 2 M thiourea, 2% w/v CHAPS, 2% w/v 3-(decyldimethylammonio)-propane–sulphonate inner salt (SB 3–10), 0.5% v/v biolytes (BioRad) and 1% v/v destreak (GE Healthcare). Samples from three separate experiments were then pooled together by mixing equal amounts of protein by mass. The pooled sample (50 μg protein) was applied to IPG strips (11 cm, pH 3–10; BioRad) and incubated overnight at a constant temperature of 20°C. After rehydration, the IPG strips were focused using the following programme: 0–500 V over 500 V h, 500–3500 V over 3500 V hs, and 3500 V for 90 000 V h at 20°C. IPG strips were incubated in equilibration buffer (8 M urea, 2 M SDS, 5 mM tributylphosphine, 40 mM Tris) for 20 min and separated by SDS-PAGE on a 4–20% polyacrylamide gel in Tris–Glycine. Following electrophoresis, gels were fixed for 16 h in fix buffer (40% ethanol, 10% acetic acid). Gels were then transferred to Flamingo stain solution (BioRad) for 8 h and imaged using a PharosFX fluorescent scanner (BioRad) and Quantity One and PDQuest software (BioRad).

### 2.7 LC-MS

Protein spots of interest were digested with Trypsin Gold (Promega) according to the manufacturer's instructions and were analysed on a Finnigan linear ion trap quadrupole (LXQ) mass spectrometer with a Finnigan Surveyor pump and microAS system (ThermoFisher Scientific, Hemel Hempsted, UK). Capillary chromatographic separation of 10 μL injection volume per sample was carried out on a C18 reverse-phase column (BioBasics C18, 0.075 mm×100 mm, 5 μm particle size, pore diameter 300 Å; Presearch, UK) with the following gradient: (mobile phase A: water with 0.1% formic acid, mobile phase B: ACN with 0.1% formic acid) 0–5 min 95% A/5% B; 5–45 min 95% A/5% B–40% A/60% B; 45–50 min 60%/40% B; 50–65 min 95% A/5% B. Gradient elution was applied for two solvents at a flow rate of 100 μL/min with a pre-column split giving a flow rate of ∼300 nL/min at the tip. Mass spectrometric detection was accomplished with a nano electrospray ionization source in positive ion mode at 2.1 kV spray voltage and 200°C capillary temperature. Data acquisition was carried out by the XCalibur software in a data-dependent triple play. In a first scan event, a full scan of all the ions in the trap over the mass range of 450–1600 *m/z* was conducted. The three highest *m/z* ratios over a threshold of 500 counts were automatically selected for ZoomScans (scan event 2) and 35% collision-induced dissociation (scan event 3) in the ion trap. The collected data were qualitatively evaluated utilizing the XCalibur software, detected peptides were identified using BioWorks 3.3.1 with an IPI database (ThermoFisher Scientific).

### 2.8 Statistical analysis

EC_50_ and one-way ANOVA with Tukey's post test analysis were carried out using Graphpad prism 3.0, a *p-*value of less than 0.05 was considered statistically significant.

## 3 Results

### 3.1 LPS induction of nitric oxide production and iNOS expression

**Figure 1:**
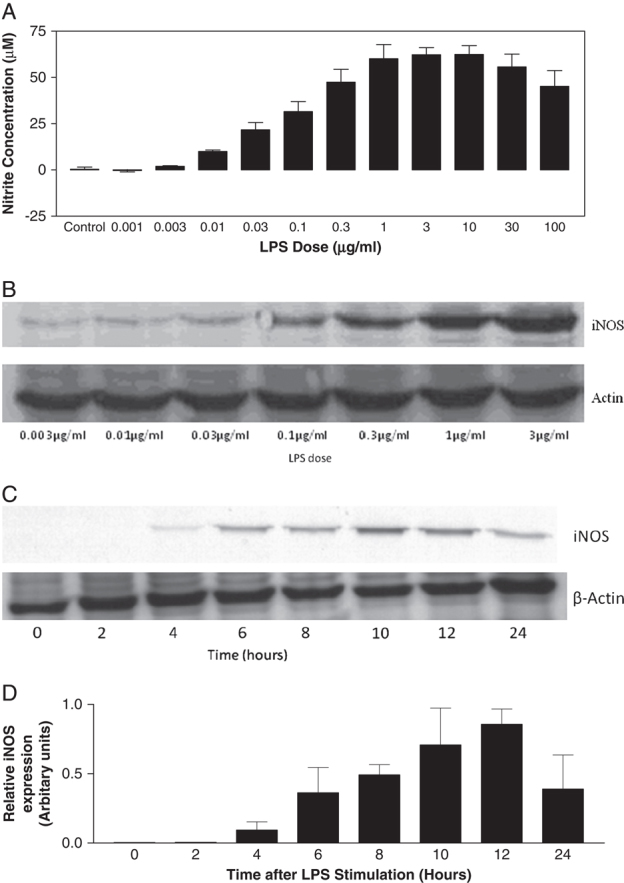
The effect of LPS dose on accumulation of nitrite in cell culture supernatant as assessed by the Griess assay (A; data are from three independent experiments (data expressed as mean±SEM)) and iNOS protein expression as assessed by Western blot analysis (B; representative blot from one of three independent experiments). LPS-induced iNOS protein expression was assessed over time (C; representative of three independent experiments) by Western blot analysis, (D) shows combined densitometry of iNOS expression from Western blot analysis (data are from three independent experiments and is expressed as mean±SEM) calculated using Quantity One software (BioRad).

To characterise the response of J774.2 macrophages to LPS, cells in culture were stimulated with concentrations of LPS ranging from 0.001 to 100 μg/mL for 24 h. The cell culture media were aspirated and analysed for nitrite content. Nitrite content in cell culture media increased at an LPS concentration of 0.01 μg/mL and increased in a dose-dependent manner, a maximal nitrite concentration of 60 μM was observed at an LPS concentration of 1 μg/mL (Fig. 1A) and this increase in nitrite concentration correlated with an increase in LPS-induced iNOS protein expression (Fig. 1B). The expression of iNOS was also assessed over 2–24 h (Fig. 1C); iNOS protein expression was not detected in unstimulated cells but was induced by LPS at 4 h after stimulation. This increased in a time-dependent manner until 10 h after which iNOS protein expression decreased but was still observable at 24 h (Fig. 1D).

### 3.2 Inhibition of LPS-induced nitric oxide production by tetracyclines

Each of the tetracyclines tested (oxytetracycline, doxycycline, minocycline and tigecycline) inhibited LPS-induced nitrite accumulation in a dose-dependent manner up to a concentration of 50 μg/mL (the highest concentration displayed no effects upon cell viability as determined by the MTT cytotoxicity assay, data not shown). Tigecycline showed the greatest activity in reducing LPS-induced nitrite accumulation at 50 μg/ml (mean=87.46±9.50 SEM % inhibition; EC_50_=3.64 μg/mL), followed by minocycline (mean=63.11±3.61 SEM % inhibition; EC_50_=14.56 μg/mL), doxycycline (mean=39.06±13.78 SEM % inhibition; EC_50_=11.99 μg/mL) and oxytetracycline (mean=20.17±4.42 SEM % inhibition; EC_50_=0.06 μg/mL). The tetracycline compound with the lowest EC_50_ was oxytetracycline however only 20.17 % was reached at the highest concentration used here and tigecycline was the most potent inhibitor of LPS-induced nitrite production, however we selected minocycline for the proteomic study as it is used more clinically and is the most studied tetracycline derivative in terms of anti-inflammatory action.

### 3.3 Proteomics of intracellular protein changes

**Figure 2:**
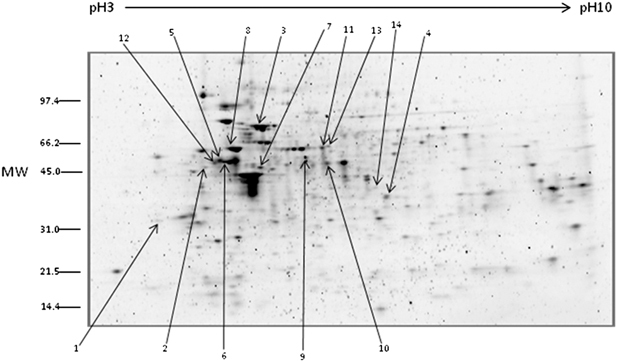
Proteins were separated on a pH 3–10 immobilised pH gradient and subsequently resolved on 4–20% gradient SDS-PAGE and stained with Flamingo total protein stain (BioRad), gels were visualised using Quantity One software and analyzed using PDQuest software. This is a representative gel showing the location of identified spots that significantly altered in density in either control versus LPS, LPS versus minocycline+LPS or control versus minocycline, spot numbers correspond to spot identification numbers in Table 1.

**Figure 3:**
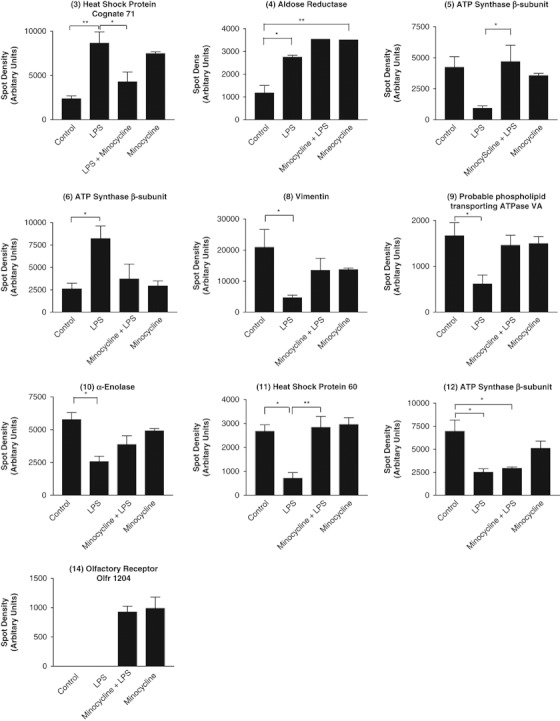
Spot density of proteins that had significantly altered expression in control versus LPS, LPS versus minocycline+LPS or control versus minocycline. Spot densities were calculated from three independently resolved gels using PDQuest software and expressed as mean±SEM. Statistical analysis was performed by an ANOVA with Tukey's post test, *p<0.05, ^**^p<0.01.

We used proteomics to investigate changes in protein expression in macrophages treated with LPS with or without a pre-treatment with minocycline (Fig. 2). Of the 14 spots identified by PDQuest analysis as having significantly changed (defined as greater than twofold increase or decrease), ten were identified by LC-nano ESI MS and we were unable to identify four protein spots. Of the ten proteins identified, eight were modulated in response to LPS (three upregulated and five downregulated), and of these LPS-induced changes two were reversed by minocycline pre-treatment, however the further three were reversed although to non-significant levels (Fig. 3, Table 1). These were mainly involved in the stress response (e.g. heat shock proteins HSP71 and HSP60) and metabolism (aldose reductase (AR) and ATP synthase). In response to treatment with LPS and minocycline, levels of five of these LPS-modulated proteins were restored, at least in part, to basal levels, and one other protein also relating to ATP synthase increased in response to minocycline compared to LPS-treated cells. Interestingly, there were also some proteomic changes in response to minocycline in the absence of LPS, heat shock protein 71 and AR, which were increased by LPS, were also increased in response to treatment with minocycline alone. Olfactory receptor 1204 expression was induced by minocycline with LPS and by minocycline alone.

**Table 1:** Proteins identified by LC-MS as altered in response to LPS±minocycline pre-treatment

Protein spot number	Accession number	Uniprot recommended name (*synonyms*)	Fold change (control versus LPS)	Fold change (LPS versus minocycline + LPS)	Fold change (control versus minocycline)	*p*-Value	Peptides identified	% coverage
1		Unidentified	2.72	NS	NS			
2		Unidentified	4.08	NS	NS			
3	IPI00323357.3	Heat shock protein 71 Cognate (*Heat shock protein 70 protein 8*)	3.67	0.50	3.17	1.1×10^−5^	TVTNAVVTVPAYFNDSQR	8.03
							DAGTIAGLNVLR	
							ARFEELNADLFR	
							FEELNADLFR	
							LLQDFFNGK	
4	IPI00223757.3	Aldose reductase (*Aldehyde reductase*)	2.33	NS	2.98	1.8×10^−8^	TIGVSNFNPLQIER	4.46
5	IPI00468481.2	ATP Synthase β-subunit, mitochondrial	NS	5.10	NS	1.7×10^−6^	FTQAGSEVSALLGR	2.55
6	IPI00468481.2	ATP synthase β-subunit, mitochondrial	3.15	NS	NS	1.5×10^−8^	LVLEVAQHLGESTVR	9.81
							LVDSGAPIKIPVGPETLGR	
							AIAELGIYPAVDPLDSTSR	
7		Unidentified	0.33	2.26	NS			
8	IPI00227299.5	Vimentin	0.24	2.90	NS	7.3×10^−13^	TYSLGSALRPSTSR	34.24
							SLYSSSPGGAYVTR	
							LLQDSVDFSLADAINTEFK	
							FANYIDK	
							ILLAELELQLK	
							ILLAELELQLKGQGK	
							EEAESTLQSFRQDVDNASLAR	
							KVESLQEEIAFLK	
							KVESLQEEIAFLKK	
							FADLSEAANR	
							FADLSEAANRNNDALR	
							ISLPLPTFSSLNLR	
							ETNLESLPLVDTHSK	
							ETNLESLPLVDTHSKR	
							DGQVINETSQHHDDLE	
								
9	IPI00338618.5	Probable phospholipid transporting ATPase VA (*ATPase class V type 10A; P-locus fat-associated ATPase*)	0.37	NS	NS	4.7×10^−5^	FRGYIMHSNGEKAGLHK	1.15
10	IPI00462072.2	α-Enolase (*2-phospho-D-glycerate hydrolyase; Non-neural enolase*)	0.44	NS	NS	1.9×10^−5^	YITPDQLADLYK	3.06
11	IPI00308885.5	Heat shock protein 60 (*60 kDa chaperonin; Chaperonin 60*)	0.27	3.98	NS	7.9×10^−10^	LVQDVANNTNEEAGDGTTTATVLAR	7.12
							VGLQVVAVK	
							LSDGVALK	
12	IPI0046841.2	ATP Synthase β-subunit, mitochondrial	0.32	NS	NS	2.3×10^−8^	LVLEVAQHLGESSTVR	7.27
							FTQAGSEVSALLGR	
							LVPLKETIK	
13		Unidentified	N/A	Not detected in LPS treatment	Not detected in control			
14	IPI00353314.5	Olfactory Receptor Olfr 1204	N/A	Not detected in LPS treatment	Not detected in control	3.8×10^−4^	PAATLPIDKAVALFYTMITPMLNPLIYTLR	9.63

Summary of proteins identified by LC-MS analysis protein spots excised from two-dimension gels. Xcalibur 2.0 and Bioworks 3.1 software was used to identify proteins from LC-MS data. Fold change reported if statistically significantly altered (*p*<0.05), NS=not significant.

## 4 Discussion

The J774.2 cell line responded to LPS by producing nitric oxide in a dose-dependent manner (Fig. 1A), this was associated with a dose-dependent increase in iNOS protein expression (Fig. 1B). The lowest concentration of LPS that produced a significant level of nitrite was 0.01 μg/mL. LPS-induced iNOS protein expression was maximal at 10 h post-stimulation and decreased by 24 h (Fig. 1C), suggesting that iNOS had been induced and subsequently decreased in expression within the 24-h experimental period in response to 0.01 μg/mL LPS.

Each of the tetracyclines inhibited LPS-induced nitric oxide production. Inhibition was associated with an inhibition of iNOS expression, the protein responsible for producing nitric oxide (data not shown). These results suggest that the inhibitory effect was due to inhibition at a transcriptional or translational level and that tetracyclines had no direct effect upon the activity of the iNOS protein. Each of the tetracyclines inhibited the production of nitric oxide in a dose-dependent manner. The differences in potency suggest that either they have different activities upon the targets responsible for this action or they possess different uptake characteristics. In terms of antimicrobial efficacy, tetracyclines have different affinities for the 30S bacterial ribosomal subunits resulting in different therapeutic efficacies 27. Little information is available on the uptake and release of tetracyclines by mammalian cells 28, 29 and no data are available for uptake by J774 cells. This is the first report to show that the new tetracycline compound tigecycline is able to inhibit LPS-induced nitric oxide production and release from macrophages. Tigecycline was the most potent of the tetracyclines tested in terms of inhibition of LPS-induced nitric oxide production and warrants further investigation for its anti-inflammatory properties.

In this study, the effects of minocycline upon the J774 proteome were investigated and a number of significant changes in levels of protein expression were detected. One limitation with proteomic information is that although changes in expression or post-translational modification of a protein can be detected, the activity of proteins is not assessed, it therefore cannot be ruled out that a drug treatment does not functionally restore a LPS-induced alteration without affecting the level of protein expression. Below is a brief discussion of some of the proteins identified here as being altered by LPS in the presence or absence of minocycline pre-treatment and their potential effect on inflammation or the anti-inflammatory action of minocycline.

Heat shock proteins (HSPs) are molecular chaperones that maintain the structural integrity of proteins. Molecular chaperones direct the folding and unfolding of proteins in cellular processes and are involved in sensing cellular stress from environmental or pathological stimuli, upon sensing serious misfolding of proteins or protein aggregation, molecular chaperones can initiate apoptosis 30. The increased expression and in some cases cellular release of HSPs in response to LPS stimulation has been observed previously 31. HSPs act as cytokines and can cause apoptosis in neighbouring cells. HSP60 has been shown to act as a danger signal for the immune system and to initiate a strong pro-inflammatory response in the immune system of both humans and mice 32. The upregulation of both HSP60 and HSP70 in response to LPS has been well documented 33, 34; however, the two HSPs identified here showed different patterns of expression, HSP60 was downregulated in response to LPS whereas HSP71 was upregulated in response to LPS.

AR mediates the metabolism of glucose to sorbitol utilizing NADH as an enzymatic co-factor in the polyol pathway; the activity of AR has previously been associated with secondary complications with diabetes. As well as metabolizing components of the glucose pathway AR can also reduce 4-hydroxy-trans-2-nonenal, a toxic product of lipid peroxidation 35, and although the mechanism is unclear, AR appears to reduce the level of glucose-induced oxidative stress. AR also has an impact on the production of nitric oxide and inhibiting AR expression or activity caused a reduction in LPS-induced nitric oxide production and subsequent death 36. However, this effect was not observed here, and nitric oxide was produced despite AR also being expressed. During an inflammatory response it is the function of macrophages to kill invading pathogens and remove toxic or potentially toxic intermediates. The increased expression of AR may be a protective mechanism of macrophages to survive the toxic intermediates they produce and release to kill pathogens. Inevitably peroxynitrite (ONOO^−^), the product of nitric oxide and superoxide radicals that macrophages produce, are damaging to the surrounding host tissue and themselves, the expression of AR is at least in part able to detoxify products of lipid peroxidation. The functional status of AR is not clear from the proteomic data and therefore although we observe that AR levels are increased, we cannot rule out that under these conditions AR function is diminished. The inhibition of AR has been shown to be beneficial in a number of inflammatory diseases including diabetes and cardiac and sepsis 37, 38. AR requires NADH as a co-factor to function, a proposed mechanism of action of minocycline is to act as an NADH mimetic and inhibit enzymatic function of poly-(ADP)ribose polymerase 18.

ATP synthase is a protein composed of 16 subunits encoded by both nuclear and mitochondrial DNA and is involved in maintaining the energy requirements of the cell. The subunit identified here is the β subunit produced from mitochondrial precursor mRNA. The β subunit is an important component of the ATP synthase protein and is responsible for binding co-factors involved in ATP synthesis 39. Three protein spots that were described as altering in response to LPS and/or minocycline were identified by nano-LC-ESI-MS as the β subunit of ATP synthase. These spots that were all identified as the β chain of ATP synthase were at similar molecular weights but had separated with different p*I* values, probably indicating that they have different post-translational modifications. Post-translational modifications of ATP synthase that have been described previously include phosphorylation and nitrosylation 40, 41. It has been reported that in type two diabetes, ATP synthase β chain is phosphorylated which results in a basic shift in p*I* 42, and it has been suggested that phosphorylation results in decreased activity of ATP synthase.

Vimentin was observed here as being reduced in response to LPS, which is subsequently restored by minocycline+LPS treatment. Vimentin is an intermediate filament protein that is cleaved in response to caspases activated by apoptotic and macrophage activation processes. LC-MS data show that the vimentin protein spot identified here corresponds to the full-length protein. We therefore could be observing protease-mediated cleavage of vimentin in response to LPS treatment, which is inhibited by a minocycline pre-treatment. The function of secreted or cell surface expressed vimentin is unclear but is associated with ageing and atherosclerosis, and potentially enhances a pro-inflammatory phenotype; vimentin secretion has been observed by activated macrophages in a protein kinase C (PKC)-dependent mechanism. Reduced cellular expression of vimentin could be due to increased secretion; minocycline has previously been shown to inhibit PKC activation in response to LPS stimulation and this could be a mechanism of inhibiting vimentin secretion.

In conclusion, we present data here to show that three clinically important tetracyclines: oxytetracycline, doxycycline, minocycline and the modified tetracycline (glycylcycline), tigecycline, inhibit LPS-induced nitrite accumulation in the J774 murine macrophage cell line. This inhibition is achievable, albeit to differing maximal amounts, by all of the compounds tested suggesting that it is a property shared by all tetracycline drugs and that the structure–activity relationships should be investigated further to maximise the anti-inflammatory actions of tetracyclines. This is the first report to show the inhibitory effect of tigecycline on LPS-induced nitrite accumulation suggesting that it may therefore be the drug of choice in sepsis where anti-inflammatory therapy is required alongside anti-bacterial therapy.

Proteomic analysis of the J774 cell line in response to LPS with or without a minocycline pretreatment identified a number of changes in protein expression and post-translational modifications. The data showed that although some of the LPS-mediated proteomic changes were reverted to control levels by minocycline, some were not suggesting that minocycline does not inhibit complete macrophage activation and that a number of LPS-induced functions are not affected by minocycline. More studies are required to fully elucidate the molecular mechanisms involved in the anti-inflammatory role of minocycline, but this report identifies novel effector molecules that are worth further investigation.
